# Delayed Diagnosis, Difficult Decisions: Novel Gene Deletion Causing X-Linked Hypophosphatemia in a Middle-Aged Man with Achondroplastic Features and Tertiary Hyperparathyroidism

**DOI:** 10.1155/2021/9944552

**Published:** 2021-04-15

**Authors:** Yun Ann Chin, Yi Zhao, Gerald Tay, Weiying Sim, Chun Yuen Chow, Manju Chandran

**Affiliations:** ^1^Department of Endocrinology, Singapore General Hospital, Singapore; ^2^Department of Clinical Translational Research, Singapore General Hospital, Singapore; ^3^Department of Head and Neck Surgery, Division of Surgery and Surgical Oncology, Singapore General Hospital, Singapore; ^4^Department of Pathology, Singapore General Hospital, Singapore; ^5^Complicated Metabolic Bone Disorders Clinic, Osteoporosis and Bone Metabolism Unit, Department of Endocrinology, Singapore General Hospital, Singapore

## Abstract

X-linked hypophosphatemia (XLH) is the most prevalent form of hereditary hypophosphatemic rickets associated with phosphate wasting. However, its diagnosis is often missed, resulting in patients presenting late in the course of the disease when complications such as tertiary hyperparathyroidism and renal failure have already set in. Phosphate and calcitriol replacement, both of which have undesirable consequences of their own, have historically been the main stay of therapy. We describe the case of a 57-year-old gentleman with tertiary hyperparathyroidism, who was mislabelled as having achondroplasia for many years before we made a diagnosis of XLH in him. His XLH was found to be due to a hereto unreported deletion of entire exon 14 with partial deletions of introns 13 and 14 of the PHEX gene. Perioperative management in him was fraught with surgical and medical difficulties including an operation that was technically complicated due to his multiple anatomical deformities. Our case also highlights the critical importance of timely recognition and accurate diagnosis of XLH, as well as the long-term multidisciplinary management that is needed for this disorder.

## 1. Introduction

X-linked hypophosphatemic (XLH) rickets is caused by inactivating mutations of the PHEX (phosphate regulating with homologies to endopeptidases on the X chromosome) gene. Consequently, the circulating humoral phosphaturic factor—fibroblast growth factor 23 (FGF23)—becomes elevated. The resultant hypophosphatemia leads to rickets with skeletal deformities, reduced linear growth, and metabolic bone abnormalities. More than 588 different mutations have been listed in the Human Gene Mutation Database (HGMD, http://www.hgmd.cf.ac.uk/ac/index.php, accessed on January 22, 2021). Despite XLH being the most common form of hereditary renal phosphate-wasting disorder, with an incidence of 1 in 20,000 individuals [1], it is very often under- or misdiagnosed. Though burosumab, a monoclonal antibody directed against FGF23, has recently become available in some parts of the world for the treatment of XLH [2], historically, patients with this disorder have been treated with phosphate and calcitriol replacement—a therapy with several undesirable consequences. Here, we describe the case of a patient with XLH who was misdiagnosed for years as having achondroplasia. His XLH was secondary to a novel, hereto unreported, complete deletion of entire exon 14 and partial deletions of intronic regions 13 and 14 of the PHEX gene. His case was complicated by the severe restrictive anatomical deformities he had, by the development of tertiary hyperparathyroidism and renal failure, all of which necessitated a complex decision-making process and made for a technically challenging parathyroidectomy and complicated postoperative course.

## 2. Case Presentation

**Figure 1:**
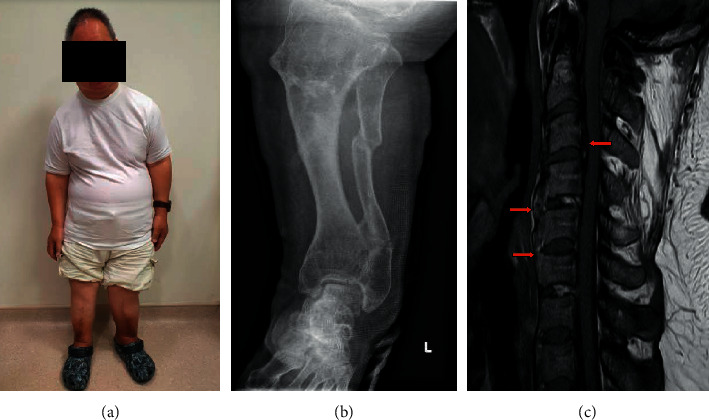
(a) Short stature, (b) deformities of the lower limb with recent fibular fracture, and (c) MRI cervical spine showing ossification of the anterior longitudinal ligament from C3 to C6 and posterior longitudinal ligaments from C2 to C4 (arrows).

The patient is a 57-year-old Chinese gentleman seen by us in July 2019. He had valgus deformity of bilateral lower limbs requiring bilateral osteotomy of the tibiae and fibulae and corrective osteotomy of the right femur all before he was 24 years old. He also had lumbar spine stenosis that necessitated decompressive spine laminectomy at the age of 51 years. He had decay of multiple teeth since he was young and had also been diagnosed as having sensorineural hearing loss and enthesopathy. On review of his medical records, it was apparent that he had hypophosphatemia since young, and he had been intermittently prescribed oral phosphate and calcitriol. His compliance to oral phosphate was quite limited due to frequent diarrhoea while on it. Compliance to calcitriol also had not been optimal. Due to his short stature of 127 cm and deformed limbs (Figure 1), he had been labelled as having achondroplasia all through his younger years.

**Figure 2:**
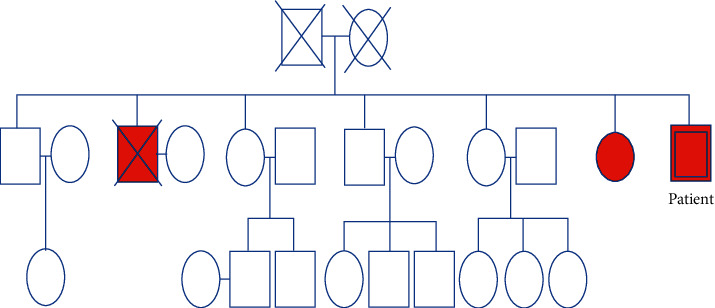
Family pedigree of the patient showing affected individuals.

There had been no evaluation performed for the aetiology of hypophosphatemia until 6 years ago when he presented initially to our department, whereupon significant phosphaturia was demonstrated with low maximum tubular capacity of phosphate (TmP) per unit volume of glomerular filtrate (TmP/GFR) of 0.24 mmol/L (0.85–1.35) calculated using the nomogram of Walton and Bijvoet [3]. He did not have hypercalciuria, albuminuria, uricosuria, or aminoaciduria. A detailed family history obtained at that time revealed that his parents were of nonconsanguineous marriage, of normal height, and with no obviously stated history of hypophosphatemia. There was a history of short stature and hypophosphatemia in his elder brother (deceased at the age of 50 years due to unknown reasons) and in a sister. This sister had a parathyroidectomy done at the age of 53 years for tertiary hyperparathyroidism. Her operative report stated that just a single adenoma was removed in her. Given this strong family history (Figure 2), the strong possibility that our patient was suffering from a hereditary hypophosphatemic disorder was raised.

Unfortunately, he defaulted subsequent follow-up and did not present to the metabolic bone clinic in the endocrine department till 6 years later at the age of 57 in July 2019. Over the last 6 years, he had developed hypercalcemia with progressively increasing serum intact parathyroid hormone (iPTH) and worsening renal function. He was still intermittently taking oral phosphate and calcitriol. His laboratory values are shown in Table 1. The phosphaturia demonstrated earlier was found to be still present. Serum FGF23 level measured by the immunometric enzyme assay was elevated at 371 RU/ml (≤180 RU/ml).

Due to the severe restrictions in positioning him on the scan table secondary to his anatomical deformities, only a dual-energy X-ray absorptiometry (DXA) scan of the 1/3^rd^ radius could be done, and this showed extremely low BMD with a T-score of −5.2 (ethnic and gender-specific reference). Based on his family history, long-standing hypophosphatemia and skeletal deformities since childhood, and his current clinical, biochemical, and radiological characteristics, the working diagnosis at this point was that he had a hereditary phosphaturic, hypophosphatemic disorder mediated by FGF23 and complicated by tertiary hyperparathyroidism and renal impairment. Whilst admitted for his workup, he had a fall and sustained a low-trauma left fibular fracture. In view of his likely tertiary hyperparathyroidism, compromised skeletal health, and worsening renal impairment, it was decided that the best option for him would be parathyroidectomy. Parathyroid imaging is usually unnecessary in tertiary hyperparathyroidism associated with long-standing renal failure since bilateral neck exploration and total parathyroidectomy with autotransplantation are the recommended operative procedures. The postoperative course after this type of surgery is usually complicated by the development of hypocalcemia that is often accompanied by hypophosphatemia and hypomagnesemia, i.e., hungry bone syndrome (HBS). The latter would be a devastating complication that our patient could ill-afford given his underlying phosphate-losing disorder. As minimally invasive of a surgery as possible was also preferable in him given his severe anatomical deformities that included an extremely short neck and restricted neck movements. Because of these two reasons, we decided to have the patient undergo parathyroid imaging in the hope that if a circumspect parathyroid adenoma could be identified, this could just be removed as had been possible in his sister. However, though parathyroid ultrasound scan showed a hypoechoic nodule at the posterior aspect of the middle third of the right thyroid lobe, the concomitant Tc-99m sestamibi scan did not reveal any hyperfunctioning parathyroid tissue. In view of the discordant imaging, we proceeded with the 4D computed tomography (4D CT) scan of the neck. It showed 3 indeterminate nodules in the visceral space, but none of them demonstrated the classic definite hypervascular pattern of enhancement classically seen with parathyroid adenomas on 4D CT imaging.

Extensive multidisciplinary discussions were held between the metabolic bone specialist, radiologist, and the head and neck surgeons on the best surgical approach and perioperative management for the patient.

**Figure 3:**
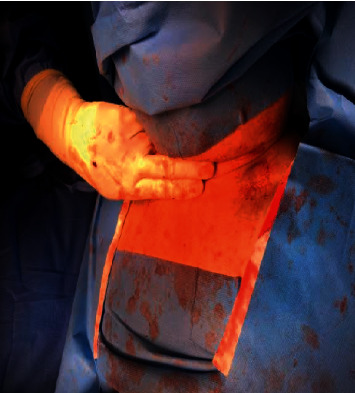
The patient's short neck as indicated by the span of <2 fingerbreadths.

He underwent bilateral neck exploration in July 2020. The operation was technically difficult with limited access due to the inability to extend the patient's rigid neck (Figure 3). The metabolic bone specialist was also in attendance in the operating room to help with intraoperative decisions.

**Figure 4:**
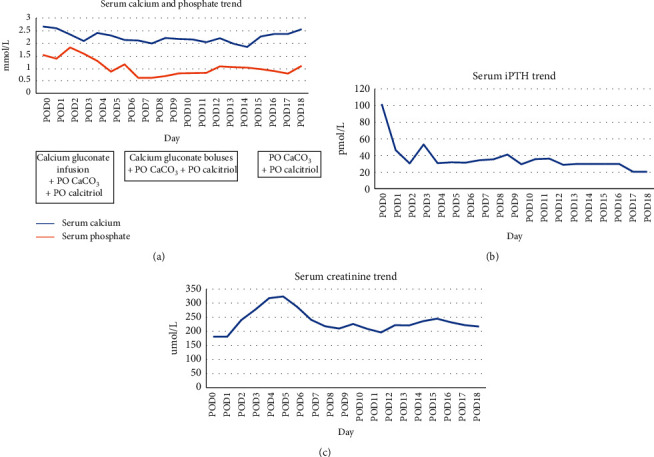
Laboratory trend after parathyroidectomy. (a) Serum calcium and phosphate trend. (b) Serum iPTH trend. (c) Serum creatinine trend.

During the surgery that lasted 3 hours, three grossly enlarged and abnormal parathyroid glands were identified and removed. After a discussion between the metabolic bone specialist and the surgeon, it was decided that a relatively normal-looking gland would be left in situ to minimize the risk of postoperative hypocalcemia. An intraoperative iPTH assay did show a decrease from a baseline value of 275 pmol/L to a level of 154 pmol/l at 10 min. This less than 50% drop at 10 minutes was expected given his significant underlying renal dysfunction. Despite the attempts intraoperatively to minimize it, he did develop HBS postoperatively. He needed continuous intravenous calcium gluconate infusion for 4 days with concurrent high dose of oral calcium carbonate (CaCo3) and calcitriol (Figure 4). His postoperative stay was complicated by respiratory failure and acute on chronic renal failure. He was discharged eventually after 19 days of hospitalisation.

**Figure 5:**
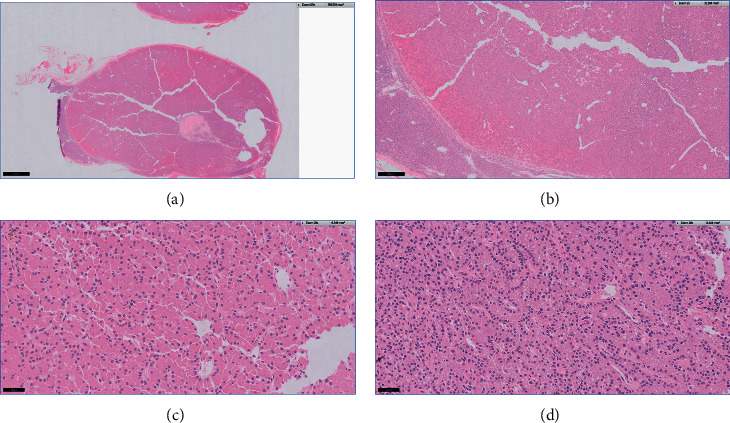
Section showing nodular proliferation of chief cells and oxyphilic cells. The component cells show a palisaded arrangement around blood vessels. (a) Low power. (b) Medium power. (c) High power (H&E, ×20): oxyphilic cells show round-to-ovoid nuclei with abundant granular eosinophilic cytoplasm. (d) Chief cells (H&E, ×20): chief cells show round nuclei and amphophilic cytoplasm. No nuclear pleomorphism or increased mitotic activity is seen.

Histology from the parathyroidectomy showed parathyroid hyperplasia in all resected specimens (Figure 5).

**Figure 6:**
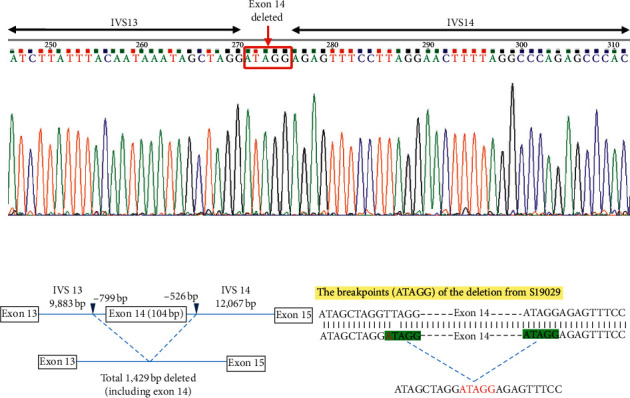
PHEX gene deletion around exon 14 and the breakpoint (ATAGG) of the deletion.

For genetic analysis, genomic DNA was extracted from whole EDTA blood via an automated approach using Maxwell® 16 Blood DNA Purification Kit (Promega, USA) in accordance to the manufacturer's protocol. The exons of the PHEX gene of interest were amplified by polymerase chain reaction (PCR) using specific design primers and DNA polymerase kit (Thermo Scientific) under the conditions as illustrated in Table 2. The PCR mix had a total volume of 25 ul, containing 1x Taq PCR buffer, 20 ng of genomic DNA, 0.4 uM of each specific forward and reverse primer, 8% DMSO, and 0.2 mM dNTP, as well as 1.5 mM MgCl. PCR products were purified using Exonuclease I and FastAP Alkaline Phosphatase (Thermo Scientific) according to the manufacturer's protocol. These purified PCR products were then ready to be sequenced using BigDye® Direct Sanger Cycle Sequencing Kit (Applied Biosystems, Foster City, Calif., USA) with post-cleanup. The specific sequences were obtained through the Sanger sequencing approach using the ABI PRISM 3500 genetic analyser (Applied Biosystems, Foster City, Calif., USA) and compared against the NCBI Nucleotide Bank (NCBI reference sequence: NG_007563.2, 144846–144869) using sequence alignment. To confirm the PCR conditions in amplifying the interests, a normal healthy individual was included in the screening. Primers were redesigned in the case of no amplification of interest. Under the same PCR conditions, for PHEX exon 14, PCR product was present in the healthy individual but not in the patient. Therefore, the screening region revolving PHEX exon 14 was expanded. The primers for PHEX exon 14 were redesigned at a position further upstream and downstream of PHEX IVS 13 and 14 regions. The final sequences of the designed primers to screen for PHEX exon 14 are shown in Table 2. A novel, previously unreported pathogenic deletion of entire exon 14 (104 bp) as well as partial deletions of IVS13 and IVS14 of the PHEX gene was observed, confirming XLH (Figure 6).

FGFR3 gene analysis did not show any mutation, indicating that he had no genetic mutation suggestive of achondroplasia.

After discharge, he continued to require CaCO_3_ and calcitriol supplementation to maintain serum calcium at low-normal levels. Phosphate supplementation had to be restarted. His kidney function stabilised. Though his serum iPTH remains elevated at 46.4 pmol/l, it has improved significantly from his baseline levels.

## 3. Discussion

The PHEX gene, which is located on X chromosome Xp22.1, is composed of 22 exons spanning 243 kb and encodes for 749 amino acid proteins [5]. PHEX mutations can be in the form of nonsense, deletions, duplications, insertions, deletional insertions, splice site, and missense mutations [6, 7]. In our patient, a single nucleotide substitute at intron 13 of the PHEX gene (position IVS13, c.1483-804T >A) was seen. The variant created an upstream breakpoint ATAGG, the pentanucleotide then likely interacted with the same pentanucleotide ATAGG downstream at IVS 14, c.1586 + 521 that then elicited abnormal splicing causing a long nucleotide deletion. This deletion involved 1,429 bp including entire exon 14 (104 bp) as well as part of IVS13 and IVS14 (Figure 5). This deletion resulted in amino acid coding frameshift starting from exon 15. This deletion has not been reported previously.

XLH usually presents in infancy or early childhood. Relative unawareness of the disease amongst physicians and orthopaedic surgeons has resulted in patients often being misdiagnosed as having achondroplasia, an autosomal dominant form of short-limbed dwarfism due to mutations of the FGFR3 gene. Other causes of hereditary hypophosphatemia such as autosomal dominant hypophosphatemia, hypophosphatemic rickets with hypercalciuria, Dent's disease, and Fanconi's syndrome were obviously unlikely in our patient with his genetic and clinical features that were typical of XLH [8].

Management of XLH has traditionally included phosphate and calcitriol replacement [9]. Diarrhoea is a common limiting factor to adequate phosphate replacement and compliance, as was evident in our patient's case. Phosphate replacement in XLH is associated with increases in PTH levels. It is postulated that high oral phosphate doses lower ionised calcium thereby stimulating PTH release through activation of the parathyroid gland calcium-sensing receptor [10, 11]. However, hyperparathyroidism is sometimes seen even before the onset of medical therapy. The pathophysiologic mechanism behind this occurrence is still poorly understood. It is also speculated that hyperparathyroidism in untreated or partially treated XLH could be a secondary event that compensates for impaired skeletal calcium mobilization, a probable feature of the underlying osteomalacia [12]. Elevated levels of FGF23 have also been linked to hyperparathyroidism in patients with XLH [13]. These two phenomena could explain the hyperparathyroidism that was seen in our patient who could be only considered as partially replaced with phosphate since he was noncompliant with his supplements. The insufficient and erratic calcitriol replacement that he was on also likely contributed to inadequate suppression of PTH and secondary hyperparathyroidism with subsequent quasi-autonomous functioning of the parathyroid glands, i.e., tertiary hyperparathyroidism [14].

The calcimimetic cinacalcet may have a beneficial role in XLH, and some reports have shown it to normalize calcium and to reduce PTH concentrations in patients with XLH [15, 16] with possible resultant amelioration of PTH-induced phosphaturia. Long-term cinacalcet is a costly proposition, and hence, our patient declined it. In addition, this is only a temporizing measure, and hypercalcemia and hypophosphatemia usually recur when it is stopped. Therefore, this was not really a viable option for our patient in whom the deleterious effects of tertiary hyperparathyroidism were already manifest with bone loss, fractures, and renal failure and in whom surgery was the only definitive therapy that could be offered.

The paradigm of treatment of XLH will likely undergo a dramatic shift with the widespread use of burosumab [2]. However, no long-term data currently exist regarding the effect of burosumab on hyperparathyroidism. Burosumab is not currently available or approved in Singapore, and therefore, this was not a therapeutic option in our patient.

Parathyroidectomy in patients with XLH is technically challenging. The operative field may be extremely limited due to anatomical deformities. Rigidity of the cervical spine caused by enthesopathy with resultant inability to extend the neck can make intubation and exploration of the thyroid bed difficult. This was the case with our patient too. Parathyroidectomy in XLH is associated with a high rate of recurrence or persistence of tertiary hyperparathyroidism postoperatively [17], and it is likely that many patients may need a repeat surgery over their lifetimes.

## 4. Conclusion

Our patient, who has XLH due to a novel, hereto unreported gene deletion, is an unfortunate example of how a diagnosis of a devastating phosphate-wasting disorder is often missed resulting in a presentation of the disease when it is already beset with complications. The pathogenesis of relentless continued elevations of PTH in this disorder remains largely unknown. Surgery for secondary and tertiary hyperparathyroidism in patients with skeletal, cartilage, and joint sequelae of this disease remains one of the most challenging operations for the head and neck surgeon. The management of metabolic derangements including that of hypocalcemia after parathyroidectomy in these patients with ongoing concurrent phosphate losses from the underlying disorder needs to be carefully planned and thought out. This is one situation where a close synergy between the metabolic bone specialist and the surgeon is imperative.

Consent was obtained from the patient for both obtaining photographs as well as for the case write-up.

## Figures and Tables

**Table 1:** Laboratory values over the years.

	July 2014 (initial consultation with endocrinology)	July 2019 (re-referral to endocrinology)	July 2020 (immediately prior to parathyroidectomy)	Normal range (units)
Serum calcium	2.33	2.67	2.67	2.09–2.46 mmol/L
Serum phosphate	0.39	0.96	1.40	0.94–1.50 mmol/L
Serum iPTH	41.1	117	101	0.9–6.2 pmol/L
Serum creatinine	91	148	180	54–101 umol/L
Serum alkaline phosphatase	140	179	184	9–99 U/L
Serum 1,25-dihydroxyvitamin D	43		30	18–64 pg/ml
Serum 25-hydroxyvitamin D	33.4	11.0	17.5	>30 ng/ml
Serum fibroblast growth factor 23 (FGF23)			371	≤180 RU/ml
24-hour urinary calcium	0.99	0.65		0.82–6.74 mmol/day

**Table 2:** Genomic DNA isolation and mutation screening, DNA extraction, and amplification of PHEX exons by PCR.

PCR thermocycling conditions for amplifying the PHEX gene		Forward and reverse primer sequences for screening exon 14 [4]	
Steps	Conditions
Initial denaturation	95°C for 3 minutes	Forward primer	CAACA AGTAGGTGAC TGTCGAGCC (NG_007563.2, 144846–144869)
35 cycles		Reverse primer	GCTAAG CTATGAGGAC ACAAAGGC (NG_007563.2, 147203–147226)
Denaturation	95°C for 30 seconds		
^*∗*^Annealing	60°C for 45 seconds		
Extension	72°C for 60 seconds		
Final extension	72°C for 10 minutes		
Hold	4°C		

^*∗*^Annealing temperature is specific to each primer set; 60°C for screening exon 14 of PHEX.

## Data Availability

No data other than that of the particular patient whose case is being reported here were used.
